# Abstracts

**Published:** 1901-08

**Authors:** 


### ABSTRACTS.

### HYPEREMESIS GRAVIDARUM.

This is a disorder that occurs during pregnancy (Smithwick, *Southern Med. Jour.*), and in most cases is confined to the earlier stages. It may be said that nausea and vomiting are, to a certain extent, normally associated with the pregnant condition of the female, but in some instances it assumes such proportions that it not only becomes pathological, but extremely dangerous. It may vary in severity from a slight "morning sickness" to an extreme state of nausea and vomiting, so that nothing will remain in the stomach, and even the thought of food will cause retching. In some of the more severe cases, if allowed to progress, the emaciation and exhaustion soon become a matter of no small moment and, unless the trouble is brought to a termination, death may occur as a result.

Hundreds of remedies have been recommended for its alleviation, but experience teaches that in most cases the gravid uterus has to be emptied of its contents to secure relief. In other less severe the desired relief comes from less radical measures.

In the treatment of pernicious vomiting of pregnancy it is unusually the custom to put off the producing abortion, as long as possible. Of course it is desirable to save the life of the child if possible, but it is not at all desirable to sacrifice the lives of both mother and child simply to give it a chance.

Among the numerous remedies recommended in the treatment of this condition, ingluvin (Wm. R. Warner & Co., Philadelphia,) gives me the best clinical results. I have frequently seen patients who would vomit immediately upon taking anything into the stomach, almost relish this preparation and the vomiting immediately cease, irrespective of its primary cause. Having had such good results with it in its various forms of nausea and vomiting, I was induced to try it in the vomiting of pregnancy, both physiological and pathological, and have had excellent results up to date. In those patients in whom the vomiting amounts to nothing more than morning sickness, which may be considered physiological, it gives great relief; also when it comes to be pathological. It relieves the nausea and increases the appetite and assimilation to a marked degree, so that the patient's system is put in an excellent condition to undergo the ordeal of labor.

The following cases illustrate and confirm these statements:

Mrs. M., aged 22, primipara. Began suffering, presumably about the third week of pregnancy, with nausea and vomiting of rather a severe type. I administered some of the customary remedies, but she continued to grow worse, until about the tenth week, when I was seriously considering the propriety of inducing abortion. However, having had good results with the use of ingluvin in nausea and vomiting due to other causes, and associated with other conditions, I determined to test it in this case. I prescribed it in fifteen grain doses every four hours. The first dose was retained, quite to my surprise, as hitherto she had vomited everything taken into the stomach. From this time on she began to improve, being troubled only occasionally with nausea and vomiting, until she finally recovered. She gained about twelve pounds in weight and went through the remainder of the pregnancy and the period of confinement without any difficulty.

Mrs. S., aged 32. This was a third pregnancy. She had been troubled no little with nausea and vomiting during the preceding pregnancies, but at this time the condition was very greatly exaggerated. She consulted me during the sixth week, stating that there were very few times she could retain either food or drink. Her bowels were constipated, skin thick and sallow in appearance and tongue heavily coated. She was emaciated, in low spirits, as she had some difficulty with former births, all being instrumental deliveries, due to inertia of the uterus. I prescribed ingluvin in three daily doses of fifteen grains each. In one week she reported that she was improving rapidly, having only had, during that time, two spells of nausea and vomiting. Her appetite was good and she could retain almost anything that she desired for food. Her bowels were in an active condition and skin much better in appearance. Her spirits were decidedly more buoyant. I directed her to continue the medicine in the prescribed dose until near the time of confinement. She did so and I attended her. The labor was perfectly normal in all respects, and was a short one when compared with her previous labors, lasting about six hours.

Mrs. G., aged 24; second pregnancy. I was called in consultation in this case for the purpose of considering the propriety of inducing an abortion on account of pernicious vomiting. The patient was weak and exhausted, due to almost incessant retching and vomiting. She has been unable to retain any food for the last three weeks, and rectal alimentation had been resorted to; but now the rectum had rebelled, and there seemed nothing left that held out any hope for relief at all except an induced abortion. I advised the administration of ingluvin in doses of fifteen grains every three hours and very small quantities of such nourishment as she most desired. The first dose was promptly vomited, but it was immediately repeated and was retained. From this time on the medicine was retained, though her stomach was very weak for the first few days, and only very small amounts of nourishment could be retained. Once or twice during that time she had severe vomiting spells, but they eventually passed off, but she did well the balance of the term and was delivered of a healthy baby.

I frequently administer ingluvin to my patients who are in a pregnant condition and are suffering with nausea and vomiting of a mild degree, and find that it gives very great relief. They gain in weight during its administration, their systems are put in a healthy condition, and they are much better prepared and able to stand the strain of labor than those who have not taken the preparation. Ingluvin has, on every occasion, served my purpose well; indeed, far better than anything else I have ever tried.

---

### THE ANODYNE TREATMENT OF ACUTE PERITONITIS.

McCaffrey (*Medical News*) observes that the most pronounced indication for treatment in peritonitis is that for the relief of pain. Blisters and counterirritation, the older resorts, are practically useless; hot water bags and poultices are far superior, but the relief they afford is only temporary. In some cases the ice bag is more grateful than hot applications. But whether heat or cold is employed, it should be relied upon only until other lines of treatment can be instituted. Papine should be given in teaspoonful doses every hour and the doses repeated frequently enough to afford the desired results. Relief from pain, short of narcosis, should be sought, and this is generally easily obtained by proper dosage. Papine does not produce nausea, but rather prevents this symptom. In the event of the development of more or less prostration, a proper stimulant, such as strychnin or nitroglycerin, should be judiciously employed.

